# Shedding of *Staphylococcus aureus *and methicillin-resistant *Staphylococcus aureus *from adult and pediatric bathers in marine waters

**DOI:** 10.1186/1471-2180-11-5

**Published:** 2011-01-06

**Authors:** Lisa RW Plano, Anna C Garza, Tomoyuki Shibata, Samir M Elmir, Jonathan Kish, Christopher D Sinigalliano, Maribeth L Gidley, Gary Miller, Kelly Withum, Lora E Fleming, Helena M Solo-Gabriele

**Affiliations:** 1 Department of Pediatrics and Department of Microbiology and Immunology, University of Miami, Miami, Florida 33130 USA; 2 NSF-NIEHS Oceans and Human Health Center, University of Miami, Rosenstiel School for Marine and Atmospheric Sciences, 4600 Rickenbacker Causeway, EG 211 Key Biscayne, FL 33149 USA; 3 NOAA Atlantic Oceanographic and Meteorological Laboratory, Miami, FL 33149 USA; 4 Public Health and Health Education Programs, Northern Illinois University, DeKalb, IL USA; 5 Miami-Dade County Health Department, 1725 NW 167 Street Miami, Florida 33056, USA; 6 University of Miami, Department of Epidemiology and Public Health, 1120 NW 14th Street, Room 1049, Miller School of Medicine, Miami, FL 33136 USA; 7 University of Miami, Department of Civil, Architectural, and Environmental Engineering, P.O. Box 248294, Coral Gables, Florida, 33124-0630, USA

## Abstract

**Background:**

*Staphylococcus aureus *including methicillin resistant *S. aureus*, MRSA, are human colonizing bacteria that commonly cause opportunistic infections primarily involving the skin in otherwise healthy individuals. These infections have been linked to close contact and sharing of common facilities such as locker rooms, schools and prisons Waterborne exposure and transmission routes have not been traditionally associated with *S. aureus *infections. Coastal marine waters and beaches used for recreation are potential locations for the combination of high numbers of people with close contact and therefore could contribute to the exposure to and infection by these organisms. The primary aim of this study was to evaluate the amount and characteristics of the shedding of methicillin sensitive *S. aureus*, MSSA and MRSA by human bathers in marine waters.

**Results:**

Nasal cultures were collected from bathers, and water samples were collected from two sets of pools designed to isolate and quantify MSSA and MRSA shed by adults and toddlers during exposure to marine water. A combination of selective growth media and biochemical and polymerase chain reaction analysis was used to identify and perform limited characterization of the *S. aureus *isolated from the water and the participants. Twelve of 15 MRSA isolates collected from the water had identical genetic characteristics as the organisms isolated from the participants exposed to that water while the remaining 3 MRSA were without matching nasal isolates from participants. The amount of *S. aureus *shed per person corresponded to 10^5 ^to 10^6 ^CFU per person per 15-minute bathing period, with 15 to 20% of this quantity testing positive for MRSA.

**Conclusions:**

This is the first report of a comparison of human colonizing organisms with bacteria from human exposed marine water attempting to confirm that participants shed their own colonizing MSSA and MRSA into their bathing milieu. These findings clearly demonstrate that adults and toddlers shed their colonizing organisms into marine waters and therefore can be sources of potentially pathogenic *S. aureus *and MRSA in recreational marine waters. Additional research is needed to evaluate recreational beaches and marine waters as potential exposure and transmission pathways for MRSA.

## Background

*Staphyloccus aureus *is an opportunistic pathogen capable of causing a wide variety of infectious diseases and is usually associated with humans as commensal colonizing organisms in at least 30% of the population [1-3]. Staphylococcal infections are primarily of the skin and soft tissues; however, they are capable of causing much more serious systemic infections and death, especially when associated with methicillin resistance [4,5]. Initially, outbreaks of methicillin resistant *S. aureus *(MRSA) infections were associated with hospitals and healthcare-associated exposures in compromised patients; however, since the late 1990 s with the emergence of new more aggressive community-associated MRSA (CA-MRSA), these infections are no longer limited to these settings.

Since its emergence, outbreaks of CA-MRSA infections in otherwise young healthy individuals [6] have been linked to close contact and sharing of common facilities such as locker rooms, schools and prisons [7]. These community-associated strains have made their way into the hospitals [5], being responsible for a significant number of hospital-acquired infections; and in a recent report, they have now been shown to be responsible for the majority of skin infections requiring treatment in emergency departments in multiple U.S. cities [8].

Waterborne transmission routes have not been traditionally associated with *S. aureus *infections. However, in some earlier studies, investigators in Hawaii reported cases of *S. aureus *infections associated with exposure to coastal marine waters [9,10], with humans serving as the suspected primary source [11]. They also showed that these organisms are able to remain viable in seawater over several days [12]. Therefore, coastal marine waters used for recreation could provide a transmission pathway for both colonization and/or infection of individuals. Previous studies have also identified *S. aureus *in recreational marine water [12,13], and *S. aureus *and MRSA in sand [14-16].

In an earlier study attempting to quantify *S. aureus *release by humans in marine water [17], investigators showed that humans shed greater quantities of *S. aureus *than the fecal indicator bacteria enterococci. However, this earlier study was limited in its methodology and criteria used to isolate and confirm *S. aureus*, and it did not address the potential presence of MRSA in the isolates. Furthermore, the study was also limited to an adult population, and it did not evaluate for *S. aureus *colonization of the human population studied.

As recreational marine waters and beaches may be commonly used by many people over the course of a short period of time, the risk of exposure to all microorganisms that are in this environment increases. Given that transmission of *S. aureus *(including MRSA) has been documented in settings associated with shared facilities and close contact, the use of recreational marine waters and beaches could certainly represent another possible route of exposure and transmission of these potentially pathogenic organisms and warrant investigation.

The aim of this study was to evaluate the amounts, as well as the characteristics, of *S. aureus*, methicillin sensitive *S. aureus *(MSSA), and MRSA shed by humans into recreational waters and sands. In this study, *S. aureus*, MSSA, and MRSA shed from adults, and for the first time children, were identified using stringent selection and identification procedures.

## Methods

The study was approved by the Florida Department of Health Internal Review Board (IRB 1491; DOH IRB Number, H07164) and the University of Miami Internal Review Board (IRB 20070306). Consent forms were signed by each study participant (or parent/guardian), and participant identity was kept confidential. The field experimental design followed that of Elmir et al. [17,18], including the use of the same study site (a sub-tropical non-point source recreational marine beach).

### Pool field studies

The "Large Pool" field study was used to determine the total amount of *S. aureus *and the distribution of *S. aureus *relative to MSSA and MRSA released from the bodies of adult bathers [17,18]. Briefly, after filling an inflatable pool with 1400 L of local off-shore marine water (where no humans were observed swimming at the time of collection), two groups of 10 adult participants were subjected to a series of four continuous 15-minute bathing cycles conducted on a single day in July 2008 beginning at 9:00 AM and 12:00 PM, respectively. In between bathing cycles, the pool was cleaned and refilled from the same source water. Participants had no sand exposure during the first two cycles, but were exposed to beach sand during the last two cycles. Samples of the source water, pool water before participant contact (in triplicate) and pool water after participant contact (in triplicate) were collected after each cycle. Source water, pool water and residual sand samples were analyzed as described below. The demographic characteristics of the 20 adult "Large Pool" participants (10 males and 10 females) included an age range from 19 to 51 years old, and body weights ranging from 50 to 100 kg [18].

The "Small Pool" field study was used to determine the total amounts of *S. aureus *and the distribution of *S. aureus *among MSSA and MRSA released from the bodies of a pediatric population, including an estimate of the contribution from the sand adhered to the pediatric participant [18]. Briefly, in the same area of the beach as the adult studies during two days in July and August of 2008, 14 individual toddlers wearing bathing suits over diapers spent 15 to 30 minutes on the beach sand (e.g. playing, sitting, lying, walking, etc). Following sand exposure, toddlers were placed in a 190-liter tub, while local off-shore marine water (14 L) was poured from sanitized watering cans gently over their heads and bodies. When necessary the toddlers were held upright in pool by an adult with either gloved hands or hands sanitized with alcohol. Sanitation of the pool and sample collections (in triplicate) were performed as described [18]. Source water, pool water and residual sand samples were analyzed as described below. The demographic characteristics of the 14 "Small Pool" toddlers (2 males and 12 females) included ages ranging from 5 to 47 months, and weights ranging from 6.8 to 16.3 kg [18].

Prior to study initiation, nasal cultures were obtained from the anterior nares from all participants using rayon swabs (BBL culture swab: Becton, Dickinson and Company) and *S. aureus *were cultured as described below.

### Bacterial isolation and identification

**Figure 1:**
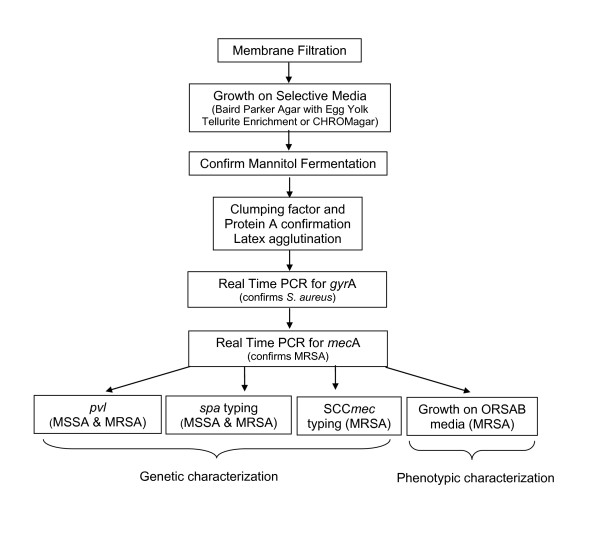
**Process Flow of Bacterial isolation and identification for *S. aureus *and MRSA**.

*S. aureus *was isolated from the water samples using a standard membrane filtration (MF) method [19], followed by growth on selective media, Baird Parker agar (Becton, Dickinson and Company, Sparks, MD) with Egg Yolk (EY) Tellurite Enrichment (Becton, Dickinson and Company), BP, and CHROMagar, CHR (Becton, Dickinson and Company) (see Figure 1 for process flow). MSSA and MRSA isolated from BP plates were subjected to genetic tests and compared to organisms isolated from nasal cultures. MSSA isolated from CHR plates was used for direct comparison of colony counts obtained during previous studies. Facilities used for processing samples were located within minutes from the study site, allowing for the processing of samples within one hour after collection. Volumes of the source water used for filtration were 10 ml and 100 ml; volumes of the pool water samples used for filtration prior to and after adult participant contact were 10 ml and 50 ml respectively; volumes of the water used for filtration after contact with the pediatric participants were 5 ml, 10 ml, and 50 ml. Multiple volumes were filtered in order to obtain quantifiable colony counts as the levels of bacteria in both the source water and the experimental pool water samples were unknown.

The analysis of *S. aureus *in sand was similar to that for water with the exception of two pre-processing steps. The first step measured the water content of sand (weight difference of sand before and after drying at 110°C for 24 h). The second step extracted bacteria from the sand particles to a predefined volume of sterile water. To accomplish this, pre-weighed un-dried sand was aseptically removed from the corresponding sample container and placed into a sterile pre-weighed jar. One hundred and ten milliliters of sterile phosphate buffer saline (PBS) were added to each jar, and the jars were shaken vigorously for 30 seconds. The samples were permitted to settle for 30 seconds, and the supernatant was subsequently used for membrane filtration. One hundred milliliters of the sand eluate samples were used for the filtration and bacterial quantification.

Following standard MF, filter membranes were placed on BP and CHR, and incubated aerobically at 37°C for a minimum of 24 h. After incubation, colonies found to be black, shiny, convex, 2-5 mm in diameter, and surrounded by clear zones (BP) or mauve (CHR), were considered presumptive *S. aureus*, and subjected to confirmatory tests. All presumptive positive isolates were transferred to Mannitol Salt agar (Becton, Dickinson and Company), for the determination of mannitol fermentation, and incubated aerobically at 37°C for 16-24 h. All mannitol-fermenting isolates were enriched [20] on Trypticase Soy Agar with 5% Sheep Blood (TSA II, Becton, Dickinson and Company) for determination of colony morphology and gross pigmentation, the ability to lyse red blood cells and to provide bacterial cells for latex agglutination tests for clumping factor and protein A using the Remel BactiStaph Latex Agglutination Test (Thermo Fisher Scientific, Lenexa, KS).

The analysis of the nasal swab cultures focused on detection and genetic characterization, rather than quantification. The method used was the same as that used for the water samples, except that the membrane filtration step was omitted. Utilizing standard aseptic techniques swabs were placed in 0.5 ml tripticase soy broth TSB (Becton, Dickinson and Company) supplemented with 6.5% sodium chloride, and incubated overnight at 37°C for enrichment. One hundred micro-liters of the overnight broth were transferred to Mannitol Salt agar (Becton, Dickinson and Company), and the organisms were identified and confirmed as detailed above.

Chromosomal DNA was extracted from colonies isolated from water, sand, and nasal cultures. Whole cell extracts were prepared from latex agglutination positive bacterial isolates using the Amplicor MTB Sputum Specimen Preparation Kit (Roche Molecular Systems, Inc., Indianapolis, IN) according to the manufacture's recommendations, and used as template for confirming and characterizing polymerase chain reactions (PCR) as outlined below. These DNA extracts (up to a maximum of 22 per filter) were subjected to PCR analysis of the *S. aureus *specific *gyr*A gene for *S. aureus *confirmation and the *mec*A gene for genetic MRSA confirmation. Oligonucleotide primers and thermal cycling conditions were used as described previously [21], with the minor modification that 5-µl of whole cell extract was used as template in initial PCR reactions instead of purified chromosomal DNA.

All organisms determined to be genotypic MRSA (testing positive for *mecA*) were re-isolated from agar plates, and grown on oxacillin resistance screening agar base media ORSAB (Remel; Thermo Fisher Scientific), a selective media for confirmation of phenotypic MRSA. All genotypic MRSA isolates from this study showed the phenotypic characteristics of MRSA. All confirmed MRSA (n = 17) and MSSA (n = 162) collected from water and sand samples and all nasal cultures were stored as stock strains at -80°C.

The number of colonies testing positive for *gyr*A gene (for *S. aureus *counts) and *mec*A gene (for MRSA counts) were reported. Counts were then adjusted to colony forming units per 100 ml water (CFU/100 ml) or per 100 g sand (CFU/100 g) using the volume of water applied to the filters or the weight of the sand collected from the pool. The numbers of microbes shed per person were determined by multiplying the difference in microbial concentrations measured before and after bathing in the pools by the water volumes corresponding to each person.

### Genetic characterization

Bacterial isolates determined to be positive for *S. aureus *specific *gyrA *and MRSA specific *mec*A were subjected to additional PCR to test for the toxin genes for Panton-Valentine leukocidin, *pvl*, to evaluate the pathogenic potential of isolated organisms as previously described [21]. Staphylococcus cassette chromosome methicillin, SCC*mec*, type was determined for all MRSA as described [22]; and Staphylococcus protein A, *spa*, type was determined for all MRSA and a representative subset of MSSA as described [23] and using RIDOM *spa *type server to analyze sequences. SCC*mec *and *spa *types were determined in order to provide specific genetic characteristics for comparison between the nasal isolates from the colonized participants and the water samples from the pools. Genetic characteristics were evaluated for all MRSA isolates and all MSSA isolates from the nasal swabs, and from the water and sand samples from the small pool. Due to the large number of *S. aureus *isolates from the large pool, genetic characterization was conducted on a representative set of the MSSA isolates from each large pool water collection and choosen to include a subset of all colony morphology, gross pigmentation and RBC hemolysis type present in each set. All MSSA collected from the small pool water samples from the single colonized pediatric participant were analyzed.

### Statistical analyses

Data analyses (including Pearson Correlations, Student T-Tests, and Sum Rank Tests) were performed using Microsoft Excel 2003 and Sigmaplot 11.

## Results

### Off shore water quality

The physical-chemical characteristics of the source water taken off shore were typical of marine waters in subtropical environments (salinity = 34 psu, pH = 7.9, temperature = 31°C). The concentrations of *S. aureus *in the source water samples prior to human exposure were primarily below the detection limit of 1 CFU/100 mL. Only 1 of 8 (13%) samples measured at the detection limit of 1 CFU/100 mL using the MF method with selection on CHR. Two of 22 (9.1%) samples measured at 10 CFU/100 mL using selection on BP. The concentrations of *S. aureus *in the pool before versus after bathing differed by two-orders-of-magnitude indicating that background levels of *S. aureus *in the source water was insignificant. MRSA was not detected from any source water samples. Overall, these results are consistent with earlier studies that showed that the offshore waters at the study site are characterized by low concentrations of viable indicator bacteria [17,18,24].

### S. aureus released by bathers

In the large pool study with adults, the total quantities of *S. aureus *released per person were lower (10^5^) by about an order of magnitude, in the first two bathing cycles as compared to Elmir et al. [17] who reported releases on the order of 10^6 ^per person (Table 1). The results appeared to converge for the last two cycles at about 10^5 ^CFU/person released. On average for all four cycles and for both groups, *S. aureus *counts were 6.3 × 10^5 ^CFU/person from BP selection which was 40% higher than 3.8 × 10^5 ^CFU/person from CHR selection.

**Table 1:** Colony forming units of *S. aureus* shed per adult

	Group I		Group II		Average		
Cycle	(BP)	(CHR)	(BP)	(CHR)	(BP)	(CHR)	(CHR)
**1**	1.3 × 10^6^	8.1 × 10^5^	*1.4 × 10^5^	BDL	7.1 × 10^5^	4.1 × 10^5^	6.1 × 10^6^
**2**	8.3 × 10^5^	8.1 × 10^5^	4.6 × 10^4^	BDL	4.4 × 10^5^	4.1 × 10^5^	3.9 × 10^6^
**3**	9.1 × 10^5^	4.2 × 10^5^	*1.0 × 10^6^	*4.3 × 10^5^	9.6 × 10^5^	4.3 × 10^5^	1.3 × 10^6^
**4**	3.6 × 10^5^	8.1 × 10^4^	*4.3 × 10^5^	*4.5 × 10^5^	3.9 × 10^5^	2.6 × 10^5^	6.8 × 10^5^

**Average**	8.4 × 10^5^	5.3 × 10^5^	4.1 × 10^5^	2.2 × 10^5^	6.3 × 10^5^	3.8 × 10^5^	3.0 × 10^6^
**Standard Deviation**	3.8 × 10^5^	3.5 × 10^5^	4.4 × 10^5^	2.5 × 10^5^	2.7 × 10^5^	7.6 × 10^4^	2.5 × 10^6^

*Water samples where MRSA was detected.

Based on analysis of water samples from a large pool with 10 adults using two different selective media, Baird Parker (BP) and Chromagar (CHR); numbers shown correspond to confirmed numbers of *S. aureus *based upon detection of the specific *gyrA *gene. BDL = below detection limit. BDLs were transformed to 1/2 of BDLs in order to estimate averages and standard deviations. The theoretical detection limit (2.8 × 10^3^ CFU/person) can be calculated from the volume of the large pool (1400 L), the largest membrane filtration volume (50 ml) and noting that 10 people bathed per cycle (adapted Elmir et al. [17]).

In the toddler studies carried out individually in the small pools, the total shedding of *S. aureus *was assumed to be the sum of the numbers observed in the sand component and in the water component. Based on the sand analysis using BP selection, the numbers of *S. aureus *transported per toddler via sand ranged from less than the detection limit (2 to 6 CFU/person) to 500 CFU/person with an estimated average of 69 +/- 145 CFU/person (Table 2). The estimated numbers of *S. aureus *(BP) shed per toddler based on the water analysis was higher, ranging from less than the detection limit (280 CFU/person) to 4.5 × 10^5 ^CFU/person, with an average of 4.3 × 10^4 ^+/- 1.2 × 10^5 ^CFU/person. The high standard deviations of the sand and water results were due to a large number of samples measured at the detection limit of the method; however, when samples were positive, the detected levels were elevated. When evaluating the significance of the sand relative to the total amount shed, the sand contributions for the single "Small Pool" bathing cycle ranged from less than 0.1 to 1.8%, with an estimated average of 0.32 +/- 0.0.09% (n = 10 subjects with sediment in the pool). Subjects were excluded from this comparison if *S. aureus *was not detected in both sediment and water samples.

**Table 2:** Colony forming units of *S. aureus* shed per toddler

Subject	Sand		Water	Ratio
**ID**	**(g)**	**(CFU per person)**	**(CFU per person)**	**(sand/water)**

**T1**	<0.1	N.D.	BDL	N/A
**T2**	6.8	500	2.7 × 10^4^	1.8%
**T3**	9.9	<6	1.1 × 10^3^	0.18%
**T4**	12.7	<6	1.3 × 10^3^	0.19%
**T5**	3.9	<6	BDL	N/A
**T6**	24.4	<6	6.3 × 10^4^	0.01%
**T7**	3.8	<6	BDL	N/A
**T8**	4.4	<6	2.5 × 10^3^	0.08.%
**T9**	6.5	<6	BDL	N/A
**T10**	8.6	160	2.3 × 10^4^	0.70%
**T11**	3.7	200	4.5 × 10^5^	0.04%
T12*	5.8	<6	1.4 × 10^4^	0.02%
**T13**	10.4	<6	2.3 × 10^3^	0.10%
**T14**	7.6	12	1.4 × 10^4^	0.09%

**Average**	7.7	69	4.3 × 10^4^	0.32%
**Standard Deviation**	5.8	145	1.2 × 10^5^	0.09%

* Indicates the study participant colonized with MSSA

N/A = Not applicable. BDL = below detection limit. BDLs were transformed to 1/2 of BDLs in order to estimate averages and standard deviations. The theoretical detection limit (2.8 × 10^2 ^CFU/person) can be calculated from the volume of water poured on the toddler (14 L) and the largest membrane filtration volume (50 ml).

### Distribution of MSSA and MRSA: Nasal Colonization and detection in water

There were a total of 34 nasal cultures (20 from the adult participants and 14 from the toddler participants). A total of 4 adult study participants were determined to be colonized with *S. aureus *by nares cultures. Two participants in group I had nasal cultures that were positive for MSSA, and two in group II were positive for MRSA. Among the adult population evaluated, the majority of the *S. aureus *shed into the water was MSSA. No MRSA was detected from Group I adults. Two of the 10 adult bathers in Group II were colonized with MRSA, and the Group II pool water was the only water where MRSA was detected. Water from the three cycles from Group II tested positive for MRSA using BP selection, and water from the two cycles were positive for MRSA using CHR selection.

Normalizing the results by the 10 adult participants in group II, MRSA shedding on a per person basis was 1.4 × 10^4 ^CFU/person for cycle 1, 7.8 × 10^4 ^CFU/person for cycle 3, and 1.0 × 10^5 ^CFU/person for cycle 4 as measured using BP selection; and 6.5 × 10^4 ^CFU/person and 9.0 × 10^4 ^CFU/person for cycles 3 and 4, respectively, for samples evaluated using CHR selection. These values represent 15 to 20% of the total *S. aureus *observed in the pool water for Group II adults.

**Figure 2:**
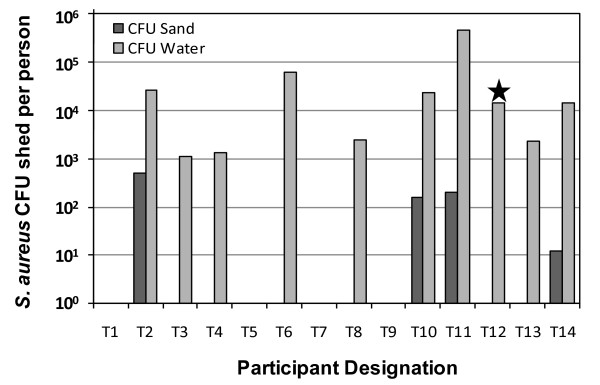
***S. aureus *CFU/person shed in small pool with individual toddlers**. Star indicates participant with MSSA colonization.

Only one of the toddlers, subject T12, was determined to have nasal colonization with MSSA; however, 10 of the 14 (71%), including T12, had *S. aureus *isolated from their water samples. Thirteen of the subjects carried sufficient sand/sediment into the pool for evaluation; however, only 4 (31%) of these were positive for MSSA, and this did not include subject T12 (Figure 2). All positive sand samples were associated with positive water samples, but only 40% (4 of 10) of the positive water samples were associated with sand; therefore, the sand did not account for the majority of MSSA shed from the toddlers not known to be colonized. In fact, the sand sample from the only toddler determined to be colonized was negative for MSSA. No nasal cultures from toddlers were positive for MRSA, and MRSA was not detected from any water or sediment samples from these participants. The lack of MRSA nasal colonization is consistent with the lack of MRSA in all of the sand and water samples from the toddler participants.

### Genetic characteristics

SCC*mec *type, *spa *type and selected gene profiles (*gyr*A, *mec*A and *pvl*) are presented for all the MRSA isolated from colonized individuals (n = 2), and water samples (n = 15) and selected toxin gene profiles and *spa *type are presented for all MSSA from colonized individuals (n = 3) and for a representative sample of corresponding water isolates (n = 17) (Table 3). Among the MRSA, the 2 organisms isolated from the participants, and 12 of 15 of the MRSA from the water samples collected from the adult Group II study were identical by these analyses. The remaining 3 MRSA differed only in *spa *type. Given the nasal culture results and comparison of the genetic analysis, the MRSA likely came primarily from the subset of colonized individuals among the 10 adult participants in the pool. Therefore, the calculated amount of MRSA shedding per person could have been up to a factor of 5 higher (assuming that MRSA came from the two participants whose nasal cultures tested positive).

**Table 3:** Bather associated *S. aureus*: MSSA and MRSA, collected as shed organisms from toddlers and adults.

Toddler Shedding: Small individual pools						
**Isolate**	**Source**	***gyrA***	***mecA***	***pvl***	**SCC*mec *type**	***spa *type**

**BLP1347**	Toddler 12 nares	pos	neg	neg	NA	t874

**BLP1275**	Toddler 12 pool	pos	neg	neg	NA	t874

**BLP1276**	Toddler 12 pool	pos	neg	neg	NA	t874

**BLP1277**	Toddler 12 pool	pos	neg	neg	NA	t411

**BLP1278**	Toddler 12 pool	pos	neg	neg	NA	t874

**BLP1279**	Toddler 12 pool	pos	neg	neg	NA	t874

**Adult Shedding: Large shared pools Group 1**						

**Isolate**	**Source**	**gyrA**	**mecA**	**pvl**	**SCC*mec *type**	***spa *type**

**BLP1207**	Group 1-Adult subject B-nares	pos	neg	neg	NA	t001

**BLP1208**	Group 1-Adult subject A-nares	pos	neg	neg	NA	t001

**BLP1295**	Group 1-cycle 1-pool	pos	neg	neg	NA	t001

**BLP1296**	Group 1-cycle 1-pool	pos	neg	neg	NA	t001

**BLP1297**	Group 1-cycle 1-pool	pos	neg	neg	NA	t001

**BLP1309**	Group 1-cycle 2-pool	pos	neg	neg	NA	t001

**BLP1310**	Group 1-cycle 2-pool	pos	neg	neg	NA	t001

**BLP1311**	Group 1-cycle 2-pool	pos	neg	neg	NA	t001

**BLP1317**	Group 1-cycle 3-pool	pos	neg	neg	NA	t001

**BLP1318**	Group 1-cycle 3-pool	pos	neg	neg	NA	t001

**BLP1319**	Group 1-cycle 3-pool	pos	neg	neg	NA	t001

**BLP1361**	Group 1-cycle 4-pool	pos	neg	neg	NA	t122

**BLP1362**	Group 1-cycle 4-pool	pos	neg	neg	NA	t122

**BLP1363**	Group 1-cycle 4-pool	pos	neg	neg	NA	t122

**Adult Shedding: Large shared pools Group 2**						

**BLP1209**	Group 2-Adult subject C-nares	pos	pos	neg	IV	t007

**BLP1210**	Group 2-Adult subject D-nares	pos	pos	neg	IV	t007

**BLP1175**	Group 2-cycle 1-pool	pos	pos	neg	IV	t001

**BLP1187**	Group 2-cycle 3-pool	pos	pos	neg	IV	t001

**BLP1189**	Group 2-cycle 3-pool	pos	pos	neg	IV	t001

**BLP1191**	Group 2-cycle 3-pool	pos	pos	neg	IV	t007

**BLP1193**	Group 2-cycle 3-pool	pos	pos	neg	IV	t007

**BLP1194**	Group 2-cycle 3-pool	pos	pos	neg	IV	t007

**BLP1195**	Group 2-cycle 3-pool	pos	pos	neg	IV	t007

**BLP1198**	Group 2-cycle 4-pool	pos	pos	neg	IV	t007

**BLP1199**	Group 2-cycle 4-pool	pos	pos	neg	IV	t007

**BLP1200**	Group 2-cycle 4-pool	pos	pos	neg	IV	t007

**BLP1201**	Group 2-cycle 4-pool	pos	pos	neg	IV	t007

**BLP1202**	Group 2-cycle 4-pool	pos	pos	neg	IV	t007

**BLP1204**	Group 2-cycle 4-pool	pos	pos	neg	IV	t007

**BLP1205**	Group 2-cycle 4-pool	pos	pos	neg	IV	t007

**BLP1206**	Group 2-cycle 4-pool	pos	pos	neg	IV	t007

Isolate designations presented with source of collection site and subject. PCR was used to determine presence of *gyrA, S. aureus *specific DNA gyrase A gene; *mecA*, methicillin resistance gene; *pvl *genes encoding Panton-Valentine leukocidin and Staphylococcal cassette chromosome *mec *type (SCC*mec*) Staphylococcal protein A type, *spa *type are shown.

Similarly, MSSA with identical *spa *types were isolated from nares cultures and corresponding waters samples for the colonized toddler and the Group I adults (Table 3). Non-identical MSSA were also isolated from water samples of the colonized toddler (one of five) and from the Group I adult study (three of twelve), indicating the presence of organisms associated with individuals but not identified in the nares cultures. MSSA was also isolated from all water collections of the adult Group II study when no individuals were identified with MSSA colonization; this also indicated the presence of organisms associated with individuals but not identified in nares cultures, and likely represents colonization of participating individuals in areas of the body other than the nares.

## Discussion

In these studies, we demonstrated that human bathers, both adults and toddlers in diapers, have the potential to release significant amounts of *S. aureus *(including MRSA) into the water column from direct shedding off their body and via sand transported on their skin. This suggests that recreational beaches may be potential exposure and transmission pathways for *S. aureus *(including MRSA). The authors hypothesize that the low background levels of MSSA in the off shore water was due to the residual effects from bather swimming activities from normal beach use given the potential persistence of these organisms in seawater [12]. These background levels, however, were very low in comparison to those levels observed during the small and large pool studies (which allowed for the quantification of the number of MSSA and MRSA released by the study participants).

The average quantities of *S. aureus *shed in this study were lower than those observed previously by Elmir et al. [17] using less stringent identification criteria. In addition to more stringent techniques, the difference in numbers may also be due to the differences in the degree to which the adults in the different studies were colonized by, and therefore shed, *S. aureus*. The shedding numbers reported above take into account the entire population, which included both those individuals who shed and those who did not shed bacteria. Therefore, individuals who participated in the large pool study who were not truly colonized, would not have contributed organisms to the pool water, yet were considered in the overall per person shedding calculations. However, when shedding was evaluated on an individual basis (as was done with the toddler study), the number of organisms shed could have been much higher per person if an adult bather in the group happened to have been colonized and was not detected by nares culture. This was the case in the adult Group II where no MSSA was isolated from participants directly, but MSSA was in the water during cycles 1 and 2 prior to sand exposure. This difference may also be due to variability of *S. aureus *shedding among different people depending upon their individual colonization status, body site colonized and quantity of organisms. Variable shedding by individuals was observed from the small pool study, where toddler shedding ranged from non-detectable levels up to values above 10^5 ^CFU/person.

Direct shedding of *S. aureus *from the body can include release from known colonized body areas such as the nares. This was seen when the large pool sample water (for Group II) was positive for MRSA only when MRSA was found in the anterior nares of participants who bathed in that water; and the majority of these organisms were shown to have the same genetic characteristics as the colonizing MRSA. Direct shedding was also observed when the single known nasally colonized toddler shed into the water sample in the small pool study. The results reported here confirm that *S. aureus *are shed by colonized adults and toddlers into the water column. This is supported by the results from both adults and toddlers in the separate pool studies. In the large pool studies, MSSA and MRSA were isolated when the participants were known only to be colonized with MRSA only (Group II); however, although only 1 toddler was shown to be colonized by nares sampling method, 10 toddlers shed MSSA. As a result of these findings, we hypothesize that both adults and toddlers are likely colonized with *S. aureus*, in particular MSSA, in other areas of the body, and that these locations contribute to bacterial shedding when exposed to water. This observation is consistent with clinical observations showing that about one third of MRSA-infected patients were not nasally colonized [25], with alternate colonization sites including skin [26] and throat [27].

Both the large pool study and the small pool study demonstrated that sand played a relatively small role in *S. aureus *shedding. In the small pool study during the single bathing cycle, sand accounted for less than 1% of shedding. Elmir et al. [18] also found that sand accounted for roughly 3.7% of the enterococci contribution in the first bathing cycle for the small pool study. For the large pool study, an increase in *S. aureus *shedding was observed when participants were exposed to sand between the second and third bathing cycles, but the impacts were less pronounced for *S. aureus *as compared to enterococci shedding as observed in prior studies [18]. Increased numbers of *S. aureus *shed in the third cycle could be associated with sand exposures; however, the ultimate source of the *S. aureus *in the sand is unknown, and may be associated with naturally existing *S. aureus *and/or from direct shedding from humans to the sand.

Because of the differences in the designs of the large pool study (adults) and the small pool study (toddlers), direct comparison of the amount of shedding between toddlers and adults in this study is limited. Nevertheless, we compared the numbers of *S. aureus *shed by adult and toddlers, keeping these limitations in mind. The average of *S. aureus *shed by adults during the four cycles in the large pool (n = 8 composites of 10 people) was 6.3 × 10^5 ^CFU/person, and by toddlers (n = 14) was 4.3 × 10^4 ^CFU/person in the small pool. In this comparison, adults shed 13 times more *S. aureus *than toddlers on average (75 times on median). It should be noted that the estimated adult body surface areas based on height and weight [28] were approximately 3.5 times larger than the toddlers in this study. After normalizing shedding numbers by the body surface factor of 3.5, the numbers of *S. aureus *shed by adults were 4 times more than toddlers on average (21 times on median). Therefore, in this investigation, toddlers in diapers shed fewer organisms than the adults; however, additional studies need to be done under the same conditions to confirm these findings.

## Conclusions

The results of this study showed that both MSSA and MRSA were shed by human populations into marine waters. The amount of shedding varied, was likely dependent upon the level of colonization of the host, and colonization was not limited to the anterior nares. In this study, the shedding of MRSA was directly dependent upon its colonization of the human host. MRSA shedding was observed intermittently, only among Group II adults and water, with the apparent lower number of humans colonized by MRSA relative to MSSA. No MRSA was observed in the sand samples as the pediatric populations evaluated in this study were apparently not colonized with MRSA. However, it is highly likely that similar studies with additional pediatric participants would result in the isolation of MRSA [29]. Future studies should focus on the collection of additional samples from human participants as the current study was limited by the restricted numbers of carriers identified. These future studies should collect samples from the skin and from other areas where *S. aureus *resides, in addition to samples from the anterior nares.

Once *S. aureus *is released from bathers, its potential for transmission is highly dependent on its persistence in the environment. Gregg and LaCroix [30] inoculated saltwater pool water with MRSA, and found very low levels after 1 hour exposure. They concluded that swimming pool water would not likely put children at risk for acquiring MRSA. However, we argue here that more research is needed to evaluate the risk of illness associated with water exposures and the potential for transmission through sand, including the residency time of these human pathogens in both recreational marine waters and beach sand [12]. Future research should be conducted with *S. aureus *species from actively colonized individuals, as the current study found large amounts released from individuals, and the actively growing clinical strains may survive differently in comparison to laboratory grown strains used for inoculation experiments. Experimentation should closely control environmental factors as some studies have documented growth of *S. aureus *under optimal environmental conditions [31].

Overall, the results from this study confirmed that both adults and toddlers can be sources of potentially pathogenic MSSA and MRSA in recreational marine waters, and support the potential for exposure and transmission of these organisms through the use of recreational beaches. More research is needed to evaluate the persistence of strains released by humans into the water column and the possibility of disease transmission at recreational beach sites.

## Authors' contributions

LRWP with ACG, CDS, MLG, and TS performed all the laboratory analyses and with SME, JK, GM, KW, HMSG, and LEF performed all the field studies. LRWP, JK, LEF, TS, and HMSG performed all the statistical analyses. All authors contributed to and edited the manuscript.
